# Analgesic efficacy of preemptive local wound infiltration plus laparoscopic-assisted transversus abdominis plane block versus wound infiltration in patients undergoing laparoscopic colorectal resection: study protocol for a randomized, multicenter, single-blind, noninferiority trial

**DOI:** 10.1186/s13063-019-3509-y

**Published:** 2019-07-02

**Authors:** Corrado Pedrazzani, Soo Yeun Park, Giovanni Scotton, Jun Seok Park, Hye Jin Kim, Enrico Polati, Alfredo Guglielmi, Gyu Seog Choi

**Affiliations:** 1 Division of General and Hepatobiliary Surgery, Department of Surgical Sciences, Dentistry, Gynecology and Pediatrics, University of Verona, Verona, Italy; 2 Colorectal Cancer Center, Kyungpook National University Medical Center, School of Medicine, Kyungpook National University, Daegu, Korea; 3 Anesthesia and Intensive Care Section, Department of Surgical Sciences, Dentistry, Gynecology and Pediatrics, University of Verona, Verona, Italy

**Keywords:** Colorectal surgery, Laparoscopy, Postoperative analgesia, TAP block, Wound infiltration

## Abstract

**Background:**

Transversus abdominis plane (TAP) block and wound infiltration (WI) are common locoregional anesthesia techniques for pain management in patients undergoing colorectal laparoscopic surgery. Comparative data between these two practices are conflicting, and a clear benefit of TAP block over WI is still debated. The main purpose of this study is to determine the efficacy in pain control of WI compared with WI plus laparoscopic TAP block (L-TAP) in cases of laparoscopic colorectal resection. Secondary aims are to evaluate other short-term results directly related to pain management: the need for rescue analgesic drugs, the incidence of postoperative nausea and vomiting, the resumption of gut functions, and the length of hospital stay.

**Methods/design:**

This is a prospective, randomized, controlled, two-arm, multicenter, single-blind study evaluating the efficacy of postoperative analgesic management of WI versus WI plus L-TAP in the context of laparoscopic colorectal surgery. Randomization is at the patient level, and participants are randomized 1:1 to receive either WI alone or WI plus L-TAP. Those eligible for inclusion were patients undergoing laparoscopic resection for colorectal tumor or diverticular disease at the Division of General and Hepatobiliary Surgery, Verona University, Verona, Italy, and at the Colorectal Cancer Center, Kyungpook National University, Daegu, Korea. Fifty-four patients are needed in each group to evidence a difference greater than 1 of 10 according to the numeric rating scale for pain assessment to establish that this difference would matter in practice.

**Discussion:**

The demonstration of a noninferiority of WI compared with WI plus L-TAP block would call into question TAP block usefulness in the setting of laparoscopic colorectal surgery.

**Trial registration:**

ClinicalTrials.gov, NCT03376048. Prospectively registered on 15 December 2017.

**Electronic supplementary material:**

The online version of this article (10.1186/s13063-019-3509-y) contains supplementary material, which is available to authorized users.

## Background

In colorectal surgery, one of the major innovations of the last two decades relates to the introduction of evidence-based protocols, also summarized by the concept of enhanced recovery after surgery (ERAS) programs. ERAS programs include a set of perioperative procedures designed to reduce perioperative surgical stress. Compared with traditional protocols, ERAS is associated with a significant decrease in general and surgical complications, a faster recovery of bowel function, and a shorter hospital stay [1, 2].

In an 8-year experience with an ERAS protocol in colon cancer surgery, nonopioid analgesia was suggested to be one of the strongest predictors of a shorter hospital stay [3]. A multimodal opioid-sparing analgesic approach as oral or intravenous nonopioid analgesia, wound infiltration (WI), and neural blockade techniques is associated with a reduction of postoperative nausea and vomiting (PONV); prolonged postoperative ileus (PPOI); urinary retention; somnolence; delayed mobilization; and, in the elderly, postoperative delirium [4].

Local anesthesia (LA) is widely considered an important component of multimodal analgesia. Transversus abdominis plane (TAP) block is one such intervention that consists of the injection of LA into the plane between the internal oblique and transverse abdominis muscles in the midaxillary line, halfway between the costal margin and iliac crest, where the somatic nerves from T6 to L1 run to innervate the anterior abdominal wall layers from the skin to the parietal peritoneum. As a result, TAP block reduces pain sensation deriving from the anterior abdominal wall nociceptive stimuli. The most common methods used to deliver a reliable and reproducible TAP block are the ultrasound-guided method (U-TAP), generally delivered by the anesthesiologist under ultrasound guidance, and the laparoscopically assisted method (L-TAP), performed by the surgeon with direct laparoscopic visualization of the site of injection.

A recent meta-analysis of 13 studies, including 7 randomized controlled trials (RCTs), that evaluated the efficacy of TAP block in a homogeneous population of patients undergoing laparoscopic colorectal surgery demonstrated that TAP block is associated with a significant reduction in postoperative opioid consumption in the first postoperative day (POD) together with a faster recovery of bowel function [5]. Last, TAP block performed before surgery appears to provide better analgesia than TAP block performed at the end of the surgical procedure [6].

The guidelines for perioperative care in elective colorectal surgery recently published by the Enhanced Recovery After Surgery Society strongly recommend the use of TAP block instead of thoracic epidural analgesia (TEA) even though the level of evidence, based on small RCTs, was considered moderated [7].

Our groups have been involved in studying the efficacy of TAP block for several years. In our previous prospective nonrandomized studies, we compared the efficacy of TAP block with WI alone, which demonstrated no difference in the control of pain or in the use of rescue analgesics. The main advantage of TAP block was limited to a reduced use of opioid analgesics [8, 9]. Furthermore, in a recent randomized, single-blind, noninferiority trial, we demonstrated that L-TAP block was not inferior to the ultrasound-guided technique in the context of laparoscopic colorectal surgery [10].

### Rationale for the trial

Despite the number of studies analyzing TAP block technique, no definite results have been published in the setting of laparoscopic colorectal surgery. The demonstration of a noninferiority of WI compared with WI plus TAP block would call into question its usefulness in the setting of laparoscopic colorectal surgery. This study is designed to evaluate L-TAP, the method of TAP block that is the easiest, faster, cheapest, and most reproducible. Moreover, TAP block administration in a preemptive fashion seems to make the technique more effective.

### Study aims and objectives

The key aim of this study is to determine the efficacy in pain control of local WI compared with WI plus L-TAP in cases of laparoscopic colorectal resection. The secondary purposes are to evaluate other short-term results directly related to pain management: the need for rescue analgesic drugs, the incidence of PONV, the resumption of gut functions, the occurrence of postoperative complications, and the length of hospital stay.

## Methods/design

### Trial design

This is a prospective, randomized, controlled, two-arm, multicenter, single-blind study evaluating the efficacy of postoperative analgesic management of WI versus WI plus L-TAP in the context of laparoscopic colorectal surgery. Randomization is at the patient level, and participants are randomized 1:1 to receive either WI alone or WI plus L-TAP.

The protocol aim is to demonstrate the noninferiority of WI compared with WI plus L-TAP in terms of effectiveness of pain management. We are supposing a noninferiority limit for pain assessment measured according to the numeric rating scale (NRS) of 1 of 10 as the largest difference that is clinically acceptable, so that a difference in NRS greater than 1 would matter in practice.

**Fig. 1:**
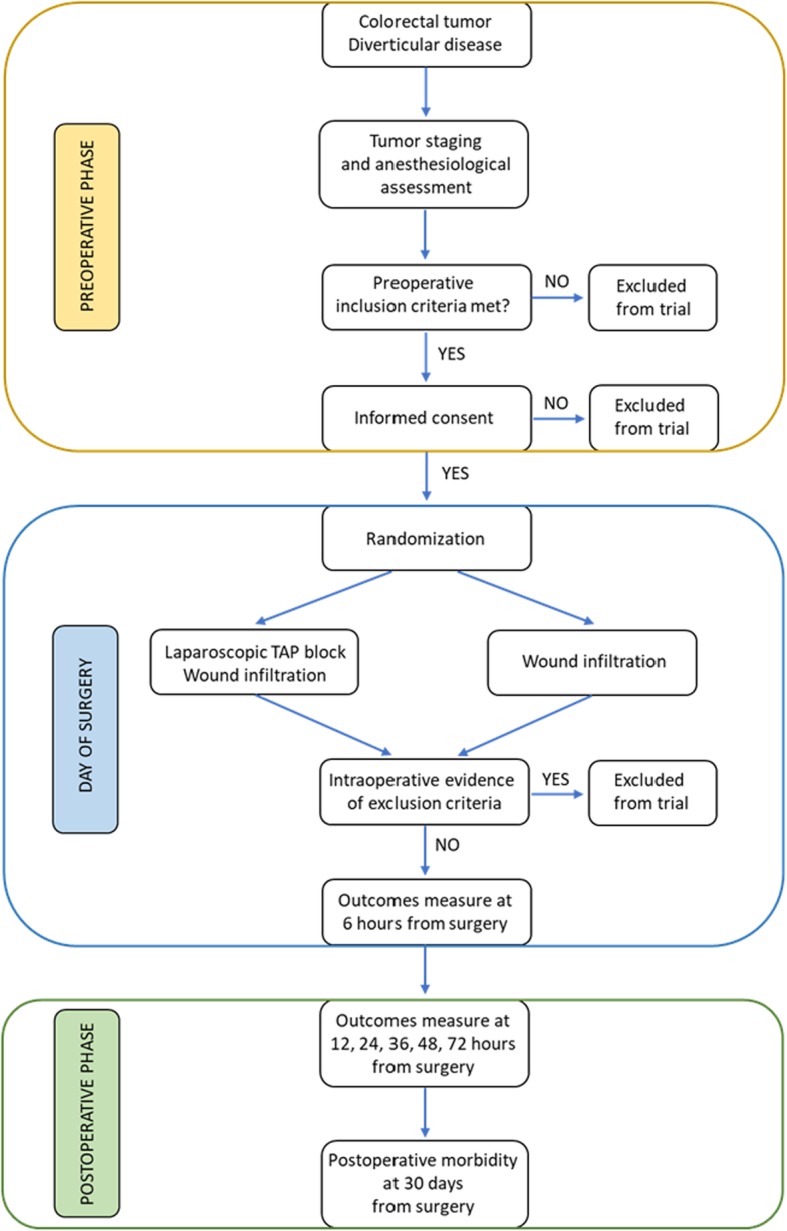
Overview study diagram

**Fig. 2:**
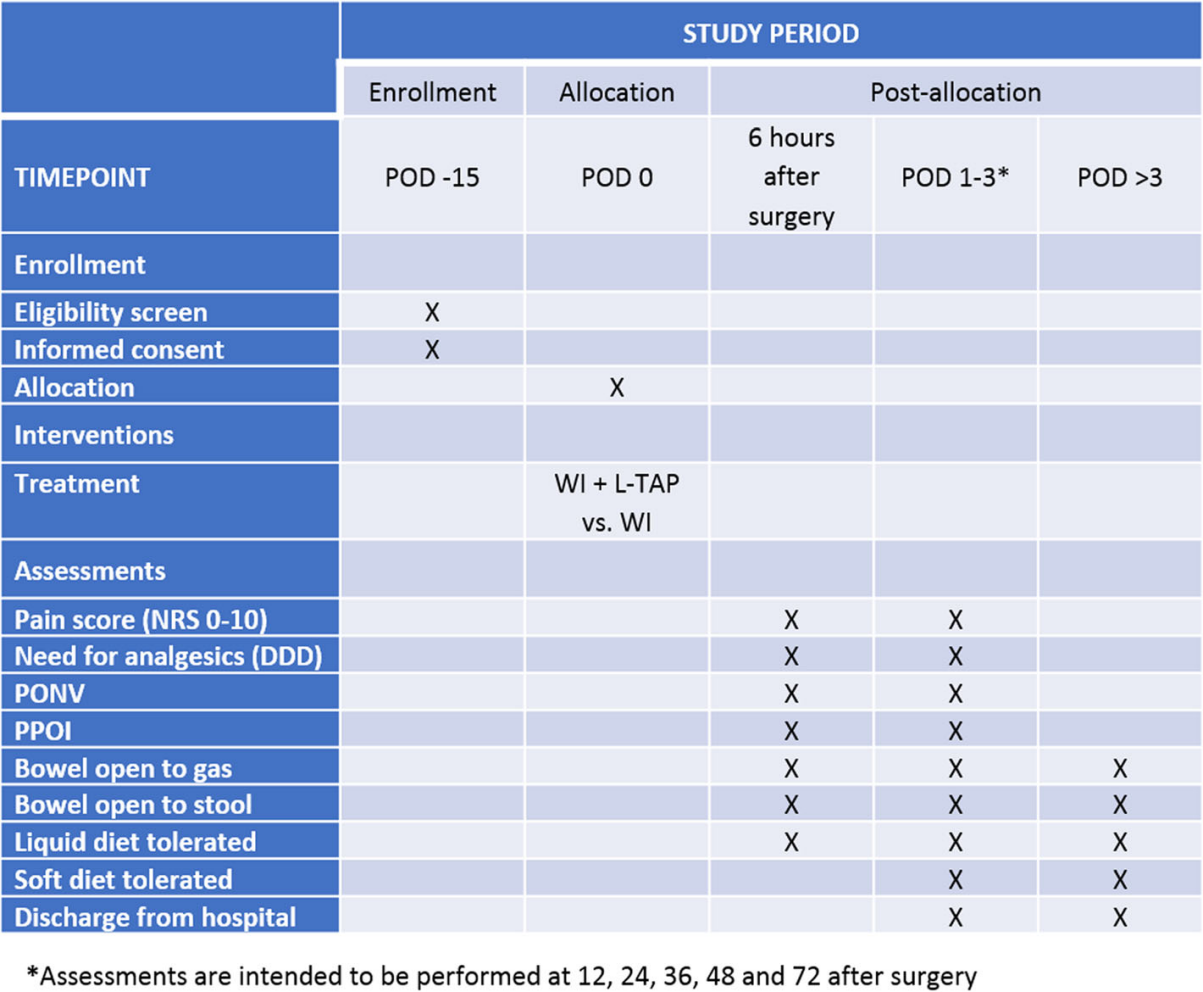
Schedule of enrollment, interventions, and assessments

Patients enrolled in this trial are asked to report their pain according to NRS at rest and during cough or movements, together with the occurrence of PONV at 6 h from surgery and twice daily until POD 3. Contemporarily, the amount of rescue analgesics is recorded, with patients being educated to request additional analgesic drugs when their pain is greater than NRS 4. Resumption of gut functions, evaluated as the passage of gas and stool as well as the ability to tolerate liquid and soft diet, is also considered together with the postoperative length of hospital stay. The study flow diagram and time of collection of outcomes are shown in Figs. 1 and 2, respectively.

This study is reported in accordance with the Standard Protocol Items: Recommendations for Interventional Trials (SPIRIT) checklist for clinical trial protocols (Additional file 1).

### Setting

Participants are recruited and operated on at the Division of General and Hepatobiliary Surgery, University of Verona Hospital Trust, Verona, Italy and at the Colorectal Cancer Center, Kyungpook National University Medical Center, Kyungpook National University, Daegu, Korea. One surgeon for each center will perform the totality of all procedures on enrolled patients. The annual caseload of laparoscopic colorectal resections for the two surgeons is approximately 200 patients.

### Study duration

The planned duration of the study is 12 months. Recruitment of patients began in April 2018 and was completed in March 2019.

### Participants

Participants are patients scheduled to undergo elective multiport laparoscopic colorectal resection for colorectal tumor or diverticular disease.

### Inclusion criteria

1. Aged older than 18 years and younger than 80 years
2. Colorectal tumor or diverticular disease as indication for surgery
3. American Society of Anesthesiologists class I, II, or III
4. Body mass index less than 35 kg/m^2^
5. Willingness to participate
6. Provision of written informed consent

### Exclusion criteria

1. Allergies or contraindication to the use of locoregional anesthesia or other analgesic drugs
2. Chronic opioid use
3. Drug or alcohol addiction
4. Severe psychiatric disorders
5. Coagulopathy; uncontrolled diabetes; severe impairment of cardiovascular, lung, or renal function
6. Need for abdominoperineal resection
7. Need for palliative surgery
8. Need for major resection other than colorectal

### Interventions

#### Induction and maintenance of anesthesia

Patients do not receive long- or short-acting sedative medication before surgery. Anesthesia is induced with short-acting agents (propofol) combined with a short-acting opioid (fentanyl or remifentanil). General anesthesia is maintained with inhalational sevoflurane or desflurane in oxygen-enriched air. Alternatively, total intravenous anesthesia with target-controlled infusion pumps and a bispectral index monitor is used. A deep neuromuscular blockade (rocuronium) is maintained with train-of-four monitoring (TOF < 3 twitch), and sugammadex is used at the end of surgery to reverse the neuromuscular blockade in order to achieve a TOF ratio > 0.9. General anesthesia entails strict glucose monitoring, maintenance of normothermia (keep body temperature > 36 °C) and a goal-directed fluid infusion. Balanced crystalloids (3–5 ml/kg/hr) is preferred to 0.9% saline and colloids, and fluid responsiveness is predicted using the stroke volume variation (with a goal of stroke volume variation < 13%).

#### Surgical technique

Colectomies for lesions located between the cecum and splenic flexure are performed using a five-port technique, and the specimen is extracted through a periumbilical incision obtained by extending the camera port or by a transverse suprapubic incision. Anterior resection, sigmoid, and left hemicolectomy are performed using a four-port technique, and the specimen is removed through a periumbilical or transverse suprapubic incision. An additional 11-mm suprapubic trocar is generally used in low anterior resection to optimize surgical field exposure. Loop ileostomy is reserved for cases in which a total mesorectal excision is performed.

#### Interventional treatment

The study is based on the concept of preemptive administration of local analgesia so that both WI and L-TAP are carried out at the beginning of the surgical procedure.

The WI group contemplates the use of a total amount of 40 ml of 0.375% ropivacaine, splitting the dose (20 ml) at the trocar and minilaparotomy sites. The infiltration is completed before each skin incision.

The total amount of anesthetic in the WI plus L-TAP group is 60 ml of 0.25% ropivacaine. First, 10 ml are used to infiltrate the trocar sites. After the positioning of the trocars and the induction of the pneumoperitoneum, bilateral TAP block under laparoscopic guidance using the “two pops technique” is carried out using 20 ml of 0.25% ropivacaine for each side. Specifically, the camera is positioned for visualization of the lateral region of the abdominal wall, and an 18-gauge laparoscopic needle is introduced under direct vision at the center of the midaxillary line between the lower costal margin and the iliac crest until the surgeon feels a “pop,” after which the surgeon injects 2 ml of normal saline to verify the correct position. At this point, after Doyle’s internal bulge sign (the bulge created when the transversus abdominis muscle with peritoneum is pushed internally) is noted, the defined amount of local anesthetic is injected. The same technique is used for the contralateral block. The remaining 10 ml are injected before skin incision at the minilaparotomy site.

#### Postoperative analgesia and PONV prophylaxis

The postoperative analgesic regimen is comparable between the two groups. Intravenous acetaminophen 1000 mg is administered three times per day (every 8 hr) from the day of surgery until POD 3. Tramadol 100 mg is prescribed as the first-choice rescue analgesic, whereas ketorolac 30 mg is used when tramadol does not achieve the desired effect.

PONV prophylaxis is based on the intravenous administration of metoclopramide 10 mg every 8 hr from the day of surgery until POD 3.

#### Withdrawal

All patients are fully informed about their participation in this study and can decide to withdraw from this trial at any time. Any information regarding patients who decide to withdraw from the protocol are excluded from the final analysis.

### Outcome measures

#### Primary endpoint

To demonstrate the noninferiority of WI compared with WI plus L-TAP for pain control in the early postoperative period after laparoscopic colorectal resection. The pain control is evaluated at 6 hr from surgery considering the greatest intensity of pain at rest and during cough, according to NRS. The NRS is graded from 0 to 10, where 0 refers to “no pain” and 10 to “worst pain.”

#### Secondary outcome measures

To demonstrate the noninferiority of WI compared with WI plus L-TAP for the other short-term postoperative outcomes that could be influenced by the analgesic regimen, we will assess the following:

1. *Pain control until POD 3*: Equal to the primary outcome, intensity of pain is evaluated according to NRS score and measured at 12, 24, 36, 48, and 72 hr from surgery.
2. *Need for rescue analgesics*: Consumption of painkillers until 72 hr was evaluated by using the defined daily dose (DDD). The DDD is a previously validated method described by the World Health Organization that converts each pain medication into a standard unit based on drug and method of administration. The conversion formula for each drug in the study was acetaminophen intravenously (1 DDD = 3 g), tramadol intravenously (1 DDD = 300 mg), and ketorolac intravenously (1 DDD = 30 mg).
3. *Occurrence of PONV*: PONV is assessed using a categorical scale graded from 0 to 2 where 0 refers to absence of nausea or vomiting, 1 to the presence of nausea, and 2 to the occurrence of vomiting.
4. *Occurrence of PPOI*: PPOI is defined as the cessation of coordinated bowel motility that prevents effective transit of intestinal contents or tolerance of oral intake lasting more than 3 days and impairing the expected postoperative course. PPOI is designed as a two-variable category, where 0 means absence of PPOI and 1 means occurrence of PPOI.
5. *Return of bowel function*: Return of bowel function is evaluated considering the time (days) needed for the bowel to open to gas and stool as well as for the patient to tolerate a liquid and solid diet.
6. *Postoperative morbidity*: Postoperative morbidity is classified according to Clavien-Dindo classification.
7. *Possibility to be discharged from the hospital*: Ability to go back home is computed considering postoperative length of hospital stay (days).

### Sample size calculation

The null hypothesis is that WI is inferior to WI plus L-TAP. To test the alternative hypothesis of noninferiority of WI compared with WI plus L-TAP, the sample size is calculated using the mean value of NRS. Considering our results with previously treated patients, the mean (standard deviation [SD]) pain intensity score according to NRS is 2.8 (1.9) for the WI plus TAP block group and 3.1 (1.9) for the WI group. These data have been used for the sample size calculation. The noninferiority limit of NRS 1 has been intended to be the largest difference that is clinically acceptable, so that a difference greater than 1 would matter in practice. A two-sided sample size calculation with 0.8 power and significance level of 0.05 reveals that 100 patients have to be included. With an estimated dropout rate of 8%, a sample size of 108 patients is required with 54 patients in the WI group and 54 patients in the WI plus L-TAP group. Patient recruitment is split between the two centers. Sample size has been calculated using PASS® version 14.0.8 with a noninferiority test for the difference of two means.

### Allocation

Patients satisfying the inclusion criteria and willing to enter the study are randomized to one of the two study arms after signing the informed consent. The randomization is performed blindly in a 1:1 fashion. The allocation to WI group or WI plus L-TAP group is achieved by creating a randomization list that includes a maximum of six subjects for nine blocks. The randomization list has been obtained by using an online program available at https://www.sealedenvelope.com/simple-randomiser/v1/lists. A separate list has been created for each center so that 54 patients are recruited at the Division of General and Hepatobiliary Surgery, University of Verona Hospital Trust, Verona, Italy, and 54 patients at the Colorectal Cancer Center, Kyungpook National University Medical Center, Kyungpook National University, Daegu, Korea. Similarly, each center collects the data separately after identifying the patients with an alphanumeric code.

### Data collection and management

A trained member from each surgical staff is in charge of data collection. Baseline demographics, as well as preoperative, intraoperative, and postoperative variables, are collected using a specifically created datasheet and stored in a specifically created dataset. The same datasheet and dataset are used in the two centers (Additional file 2).

Furthermore, to reduce as much as possible the variability among patients in reporting pain intensity, the patient is instructed on use of the NRS by the same member of the surgical staff at the time of accrual (15–20 days before surgery) and the day before surgery. To reduce the variability related to data collection, the same member will collect all other investigated variables.

### Statistical analysis

Statistical analyses will be performed on an intention-to-treat (ITT) basis using IBM SPSS Statistics software version 21.0 (IBM Corporation, Armonk, NY, USA). Because the ITT analysis includes all randomized subjects and, in this study**,** there may be up to 6% missing data, an imputation method will be established. A per-protocol (PP) analysis will also be performed. In noninferiority studies, both ITT and PP analyses are recommended and should support the noninferiority hypothesis. Continuous variables are summarized as mean and SD or as median and 95% confidence interval (95% CI) if the distribution is asymmetrical. Categorical variables are summarized as numbers and percentages. The categorical variables will be compared between groups with the chi-square test or Fisher’s exact test, depending on the number of events. The continuous variables will be compared between groups using an unpaired *t* test or the Mann-Whitney *U* test, depending on data representation. A two-sided *P* value less than 0.05 will be considered significant.

The primary outcome considers the level of pain at 6 hr from surgery graded according to the NRS. Assuming a pain score difference of 1 of 10 to be clinically significant, noninferiority is determined when the lower boundary of the 95% CI is > 1. Superiority, on the other hand, is determined when the two means are significant at the 5% level (two-sided *P* < 0.005).

The secondary objectives aim at assessing the benefits of choosing one procedure over the other. Descriptive statistics and plots will be used to compare the two groups, taking into account: (1) pain intensity scores at 12, 24, 36, 48, and 72 hr after surgery; (2) the quantity of rescue analgesic drugs consumed from the day of surgery until POD 3; (3) the occurrence of PONV; (4) the occurrence of PPOI; (5) the time needed to restore bowel function considered as the time needed to pass gas and stool as well as to tolerate a liquid and solid diet; and (6) the length of hospital stay.

## Discussion

Perioperative multimodal analgesia uses combinations of analgesic medications that act in an additive or synergistic manner to achieve pain relief with minimal or no opiate consumption. Oral or intravenous acetaminophen and nonsteroidal anti-inflammatory drugs are widely approved as the basis of this multimodal approach because they improve postoperative pain and reduce systemic opioid consumption [11]. Even if no clear benefit has been demonstrated with tramadol administration as a substitute for opioids after colorectal surgery, some authors recommend its use as one postoperative adjunct and in a rescue analgesia treatment algorithm prior to using traditional opioids [11]. TEA is considered the gold standard in patients undergoing open colorectal surgery, but it does not seem to offer any additional clinical benefits to patients undergoing laparoscopic colorectal surgery compared with alternative analgesic technique within an ERAS program [7, 12]. Therefore, current literature no longer recommends TEA for pain control after laparoscopic colorectal surgery [7, 13, 14]. TAP block and WI have been shown to efficiently control postoperative pain, reduce postoperative opioid consumption, and facilitate bowel recovery. Comparative data between these two technics are conflicting, and a clear benefit of TAP block over WI is still debated. We previously demonstrated that adding TAP block to local WI in the setting of laparoscopic colorectal surgery and the ERAS program guaranteed a reduced use of opioid analgesics and good pain control, allowing the improvement of essential aspects of enhanced recovery pathways [8, 9]. To prove the real effectiveness of TAP block, we designed a multicenter RCT comparing WI with WI plus L-TAP. Demonstration of noninferiority of WI would call into question the role of TAP block in the setting of ERAS programs.

## Trial status

Recruitment started on April 8th, 2018, and was completed on March 31st, 2019. (UniVR CESC-1509; KNUMC 2017-11-020-002).

## Additional files

Additional file 1: Standard Protocol Items: Recommendations for Interventional Trials (SPIRIT) checklist. (DOC 131 kb)

Additional file 2: Data collection sheet adopted in the two centers. (PDF 260 kb)

## Data Availability

The datasets used and/or analyzed during the current study are available from the corresponding authors (CP and YSP) on reasonable request.

## Abbreviations

CI: Confidence interval
DDD: Defined daily dose
ERAS: Enhanced recovery after surgery
ITT: Intention to treat
LA: Local anesthesia
L-TAP: Laparoscopic-assisted transversus abdominis plane
NRS: Numeric rating scale
POD: Postoperative day
PONV: Postoperative nausea and vomiting
PP: Per protocol
PPOI: Prolonged postoperative ileus
RCT: Randomized controlled trial
SD: Standard deviation
TAP: Transversus abdominis plane
TEA: Thoracic epidural analgesia
TOF: Train-of-four monitoring
U-TAP: Ultrasound-guided transversus abdominis plane
WI: Wound infiltration
