# Supplementary material for: Analgesic efficacy of preemptive local wound infiltration plus laparoscopic-assisted transversus abdominis plane block versus wound infiltration in patients undergoing laparoscopic colorectal resection: study protocol for a randomized, multicenter, single-blind, noninferiority trial
Source: Trials. 2019 Jul 2;20:391. doi: 10.1186/s13063-019-3509-y (PMC6604482; doi:10.1186/s13063-019-3509-y)
Supplement: Supplementary file 2 — Data collection sheet adopted in the two centers. (PDF 260 kb) [file 13063_2019_3509_MOESM2_ESM.pdf]

Laparoscopic ColoRectal Surgery
L-TAP-block plus Wound infiltration Vs. Wound infiltration only
2018 DATA COLLECTION FORM

PATIENT INFORMATION *(to be completed at the admission)*

Name____________________________________ **Identification code**: ______________ **DOB** *(dd/mm/yyyy):* ___ /___ /______
Sex: ❑ Male ❑ Female **Age:** ______ **Weight**: ______kg **Height** :______cm **BMI:**_______ **Smoker** ❑ Yes ❑ No
Race(s): ❑ White ❑ Black/African ❑ Asian ❑ Indian ❑ Other **Alcohol abuse** ❑ Yes ❑ No **Drug addiction** ❑ Yes ❑ No
Diabetes ❑ Yes ❑ No **Dyslipidemia** ❑ Yes ❑ No **Hypertension** ❑ Yes ❑ No **Cardiovascular Disease** ❑ Yes ❑ No
Chronic Kidney Disease ❑ Yes ❑ No **Other** ❑ Yes ❑ No *If yes specify*______________________________
ASA **score** ❑1 ❑2 ❑3 ❑ 4
Previous abdominal surgery ❑ Yes ❑ No *If yes specify*____
Chronic analgesics ❑ Yes ❑ No *If yes specify*: ❑ NSAID ❑ Opioid ❑ Paracetamol
*Specify drug and dose*____

PROCEDURE INFORMATION *(to be completed by the surgeon at the time of surgery)*

Date of Surgery *(dd/mm/yyyy):* ___ /___ /______
Indication to surgery ❑ Diverticular disease ❑ Colon cancer ❑ Rectal Cancer
Colon Cancer site ❑ Cecum ❑Ascending ❑Hepatic flexure ❑ Transverse colon ❑Splenic flexure ❑ Descending ❑Sigmoid ❑Upper Rectum ❑Lower Rectum
Metastasis ❑ Yes ❑ No *If yes specify* :_______________ **Neoadjuvant radiochemotherapy** ❑ Yes ❑ No
Procedure: ❑ Rt Hemicol ❑ Extended Rt Hemicol ❑ Lt Colectomy ❑ Lt Hemicol ❑ AR ❑LAR ❑Other______________
Stoma ❑ Colostomy ❑ Ileostomy ❑No
Contemporary visceral resection ❑ Yes ❑ No *If yes specify* :________________________________
Port number *(including optical):* ______ **Minilaparotomy:** ❑ Suprapubic ❑ Periumbilical ❑Other: ______________________
Length of minilaparotomy *(cm)* ________ **Drain(s)** ❑ Yes ❑ No **Anesthesia time** *(mins)* ______ **Surgery time** *(mins)* ________
Intraoperative complications ❑ Yes ❑ No *If yes specify* :_________________________ **Blood loss** *(ml)* ________________
Pneumoperitoneum pressure *(mmHg)* ___________. **Total CO2 volume** *(liters)* ___________
Regional analgesia ❑ TAP-block + Wound infiltration* ❑Wound infiltration** ❑ At the beginning ❑ At the end of surgery
(to be completed by the surgeon in a separated database blinded to other research collaborators)
Intraoperative analgesic drugs *(drug and dose)* ____
Intraoperative antiemetic drugs *(drug and dose)* ____

***TAP-block+WI:** 0.25% ropivacaine 20ml each side TAP (total 40ml) + WI 0.25% ropivacaine 10 ml for mini-laparotomy and 2.5 ml for each of 4 trocar incisions
****WI:** 0.375% ropivacaine 40ml (20ml for mini-laparotomy and 5ml for each of 4 trocar incision)

POST-OPERATIVE INFORMATION *(to be completed by research collaborator blinded to regional analgesia type)*

**Post-op.**	**NRS score** *(highest score between at rest or coughing)*	**PONV score***
**4-6 h**	❑0 ❑1 ❑2 ❑3 ❑4 ❑5 ❑6 ❑7 ❑8 ❑9 ❑10	❑0 ❑1 ❑2
**12 h**	❑0 ❑1 ❑2 ❑3 ❑4 ❑5 ❑6 ❑7 ❑8 ❑9 ❑10	❑0 ❑1 ❑2
**24 h**	❑0 ❑1 ❑2 ❑3 ❑4 ❑5 ❑6 ❑7 ❑8 ❑9 ❑10	❑0 ❑1 ❑2
**48 h**	❑0 ❑1 ❑2 ❑3 ❑4 ❑5 ❑6 ❑7 ❑8 ❑9 ❑10	❑0 ❑1 ❑2
**72 h**	❑0 ❑1 ❑2 ❑3 ❑4 ❑5 ❑6 ❑7 ❑8 ❑9 ❑10	❑0 ❑1 ❑2

*PONV score: 0 no nausea/vomiting; 1 nausea; 2 vomiting
** intravenous infusion of **100 mg Tramadol** (T) or **30 mg Ketorolac** (K)
*** DDD: **100 mg Tramadol** = 1/3 DDD (1 DDD= 300 mg) **30 mg Ketorolac** = 1 DDD

Laparoscopic ColoRectal Surgery
L-TAP-block plus Wound infiltration Vs. Wound infiltration only
2018 DATA COLLECTION FORM

**Paracetamol routine doses**	**Rescue doses** *(kind and dosing)***
**Day 0 (0-24 h)** ❑1 ❑2 ❑3 ❑4<br>**Day 1 (24-48 h)** ❑1 ❑2 ❑3 ❑4<br>**Day 2 (48-72 h)** ❑1 ❑2 ❑3 ❑4<br>**Day 3 (72-96 h)** ❑1 ❑2 ❑3 ❑4	**Day 0 (0-24 h)** T 100mg ❑1 ❑2 ❑3<br>K 30mg ❑1 ❑2 ***Total DDD_____<br>**Day 1 (24-48 h)** T 100mg ❑1 ❑2 ❑3<br>K 30mg ❑1 ❑2 ***Total DDD_____<br>**Day 2 (48-72 h)** T 100mg ❑1 ❑2 ❑3<br>K 30mg ❑1 ❑2 ***Total DDD_____<br>**Day 3 (72-96 h)** T 100mg ❑1 ❑2 ❑3<br>K 30mg ❑1 ❑2 ***Total DDD_____

Prolonged postoperative ileus *(lack of intestine movement more than 3 days following surgery)* ❑ Yes ❑ No
Complications: ❑SSI ❑ Intra-abdominal abscess ❑Bleeding requiring transfusion/operative hemostasis ❑Anastomotic leak
❑Other surgical ______________________________ ❑ General complications* __________________________________
❑Reoperation *(within hospital stay)* ❑ Mortality
* pulmonary, cardiac, thromboembolic

Clavien-Dindo classification: _________

Time to first flatus (days): _______ **Time to stools** (days): ______ **Time to drain(s) removal** (days): ________

Time to liquid diet (days): _______ **Time to soft diet** (days): _______ **Time to discharge** (days):________.

Sitting on chair (hours) _______ **Walking** (hours) _______

30 days Readmission ❑ Yes ❑ No

HISTOPATHOLOGICAL INFORMATION

Histologic type: ❑Adenocarcinoma ❑ Mucinous ❑ Other (*specify*): ________________________ **Grade:** ❑G1 ❑G2 ❑G3

Maximum tumor diameter: _____cm **pT:** ❑pTX ❑pT0 ❑pTis ❑pT1 ❑pT2 ❑pT3 ❑pT4 **pN:** ❑pNX ❑pT0 ❑pT1 ❑pT2

Regional lymph nodes: n° examined: _____ n° involved: _____ **pM**: ❑pM0 ❑pM1 **Stage:** ❑0 ❑1 ❑2 ❑3 ❑4

R: ❑R0 ❑R1 ❑R2 **Distance of carcinoma:** From proximal margin ______ cm; From distal margin: _____ cm;

Circumferential margin (if applicable): _____ mm **Tumour budding**: ❑Yes ❑No **Vascular invasion:** ❑Yes ❑No
